# Laennec’s approach for laparoscopic anatomic hepatectomy based on Laennec’s capsule

**DOI:** 10.1186/s12876-019-1107-9

**Published:** 2019-11-21

**Authors:** Yue Hu, Jiong Shi, Shaohe Wang, Wenjie Zhang, Xitai Sun, Beicheng Sun, Decai Yu

**Affiliations:** 1 Biobank of Nanjing Drum Tower Hospital, The Affiliated Drum Tower Hospital, School of Medicine, Nanjing University, Nanjing, Jiangsu Province, People’s Republic of China; 2 Department of Pathology, Nanjing Drum Tower Hospital, The Affiliated Drum Tower Hospital, School of Medicine, Nanjing University, Nanjing, Jiangsu Province, People’s Republic of China; 3 Hepatobiliary and Pancreatic Center & Liver Transplantation Center, The Affiliated Drum Tower Hospital, School of Medicine, Nanjing University, Nanjing, Jiangsu Province, People’s Republic of China

**Keywords:** Laparoscopic anatomic hepatectomy, Laennec’s capsule, Surgical practice, Natural gap

## Abstract

**Background:**

Although isolating Glissonean pedicles and hepatic veins are critical procedures during anatomical hepatectomy, there is no standardized approach. We propose the novel Laennec’s approach for laparoscopic anatomic hepatectomy (LAH) based on Laennec’s capsule, which serves as the anatomic landmark for LAH. The aim of this study was to elucidate that the natural gap between Laennec’s capsule and the adjacent tissues contributes to standardization of the surgical technique for LAH.

**Methods:**

Eighty-four cases were enrolled in this observable clinical trial. They underwent LAH for liver diseases. Laennec’s approach was proposed for LAH based on Laennec’s capsule. The liver tissues close to Glissonean pedicle, hepatic veins, naked area, and inferior vena cava were collected for hematoxylin and eosin, resorcinol-fuchsin staining, and immunohistochemistry.

**Results:**

The staining revealed capsule packaging of the whole liver independent of the adjacent tissues and intrahepatic vessels. A natural gap was found between Laennec’s capsule and the adjacent tissues at different sites. Laennec’s capsule served as the landmark for isolating Glissonean pedicle and hepatic veins, mobilizing the liver, and performing Hanging maneuver. Eighty-four cases underwent LAH for liver diseases using this strategy. The operation time was 277.23 min. The mean of hospital days was 9.8.

**Conclusions:**

Laennec’s approach based on Laennec’s capsule contributes to standardization of the surgical technique for LAH, and brings innovations that facilitates safe and effective liver resection under laparoscopy.

## Background

Anatomical liver resection (ALR) is widely considered a safe and effective procedure for patients with hepatocellular carcinoma (HCC). Inflow and outflow serve as the landmarks for ALR [1, 2]. However, there is no definitive approach for the hepatic vein or the Glissonean pedicles isolation, although there are three approaches for the pedicles, i.e., intrafascial, and extrafascial with or without destruction of the parenchyma [3]. The surgical technique for isolating the inflow and outflow has not been standardized due to a lack of anatomical understanding. It has become increasingly imperative to standardize ALR with the rapid spread of laparoscopic hepatectomy [4].

In 1802, Laennec first described the membrane as a structure distinct from the serosa. Couinaud established the concept of the plate system, a fibrous, thickened part of the Glissonean sheath, and demonstrated that Laennec’s capsule has no continuity with the Glissonean pedicle [5]. In 2016 Hayashi et al. revealed that Laennec’s capsule is dissociated from the Glissonean pedicle, and extends to the peripheral Glissonean pedicles [6]. In 2017 Sugioka conducted a precise histological study of the liver and showed that Laennec’s capsule is a dense fibrous layer beneath the serosa, on the surface of the bare area, Glissonean pedicle, cystic fossa, and hepatic vein [7].

The natural gap between Laennec’s capsule and the adjacent tissues is considered as the best landmark from which to approach the Glissonean pedicle and hepatic vein, mobilize the liver, and perform the Hanging maneuver during laparoscopic anatomic hepatectomy (LAH). In 2016, our group designed and standardized Laennec’s approach for LAH in 84 cases.

## Materials

### Patients

Eighty-four cases (32 males and 52 females) with benign or malignant neoplasms or hepatolithiasis underwent LAH from January 2016 to March 2018. The mean age of the patients was 55.3 years; 23 had HCC, 28 had hepatolithiasis, 22 had a hemangioma, and 4 had cholangiocarcinomas and other liver diseases. Before the hepatectomy, the lesions or stones and liver anatomy were evaluated by contrast-enhanced magnetic resonance imaging. Laennec’s approach for LAH was used during the hepatectomy by isolating the Glissonean pedicle and hepatic vein, mobilizing the liver, and conducting the Hanging maneuver. The liver tissues close to the Glissonean pedicle, hepatic veins, naked area, and inferior vena cava (IVC) were collected for hematoxylin and eosin (H&E) and resorcinol-fuchsin (R&F) staining, and immunohistochemistry was performed for smooth muscle actin (SMA) after the operation. Perioperative management was based on the regular protocol in our center, described in our previous trial [8].

The protocol was approved by the Research Ethics Committee of Drum Tower Hospital, and conformed to the ethical guidelines of the 1975 Declaration of Helsinki. Informed consent was obtained in writing from each patient.

### H&E, R&F, and SMA staining

Five liver specimens with peritoneal covering at different sites were obtained from the livers of five patients undergoing liver transplantation for liver failure, while ten specimens at different sites were obtained from partial hepatectomy for HCC and bile duct stones. The specimens were cut into 1.5 × 1.0 × 0.3 cm transverse, sagittal, or coronal sections at the naked area, hepatic portal, hepatic veins, and IVC. The tissues were fixed in neutral-buffered formalin within 10 min of surgical resection for 24 h. Continuous sections were prepared for H&E and R&F staining. Some of the sections were used for SMA immunohistochemistry. Before R&F staining, the relevant sections were deparaffinized in dimethylbenzene. The sections were stained in the solution for 1–2 h and excess stain was removed with 95% alcohol. The R&F-stained elastic fibers were violet. The EnVision method was used for immunohistochemistry staining of SMA. The primary antibody used for immunohistochemistry was monoclonal anti-human alpha 1-SMA (ZM-0003; ZSGB-BIO, Beijing, China).

### Statistics

Data are expressed as mean ± standard deviation with the range given in parentheses. Statistical comparisons were done using the *t*-test. All statistical procedures were performed using SPSS software (ver. 11.5; SPSS, Inc., Chicago, IL, USA). *P*-values < 0.05 were considered statistically significant.

## Results

### Laennec’s capsule packages the whole parenchyma independent of the adjacent tissues and intrahepatic vessels

**Fig. 1:**
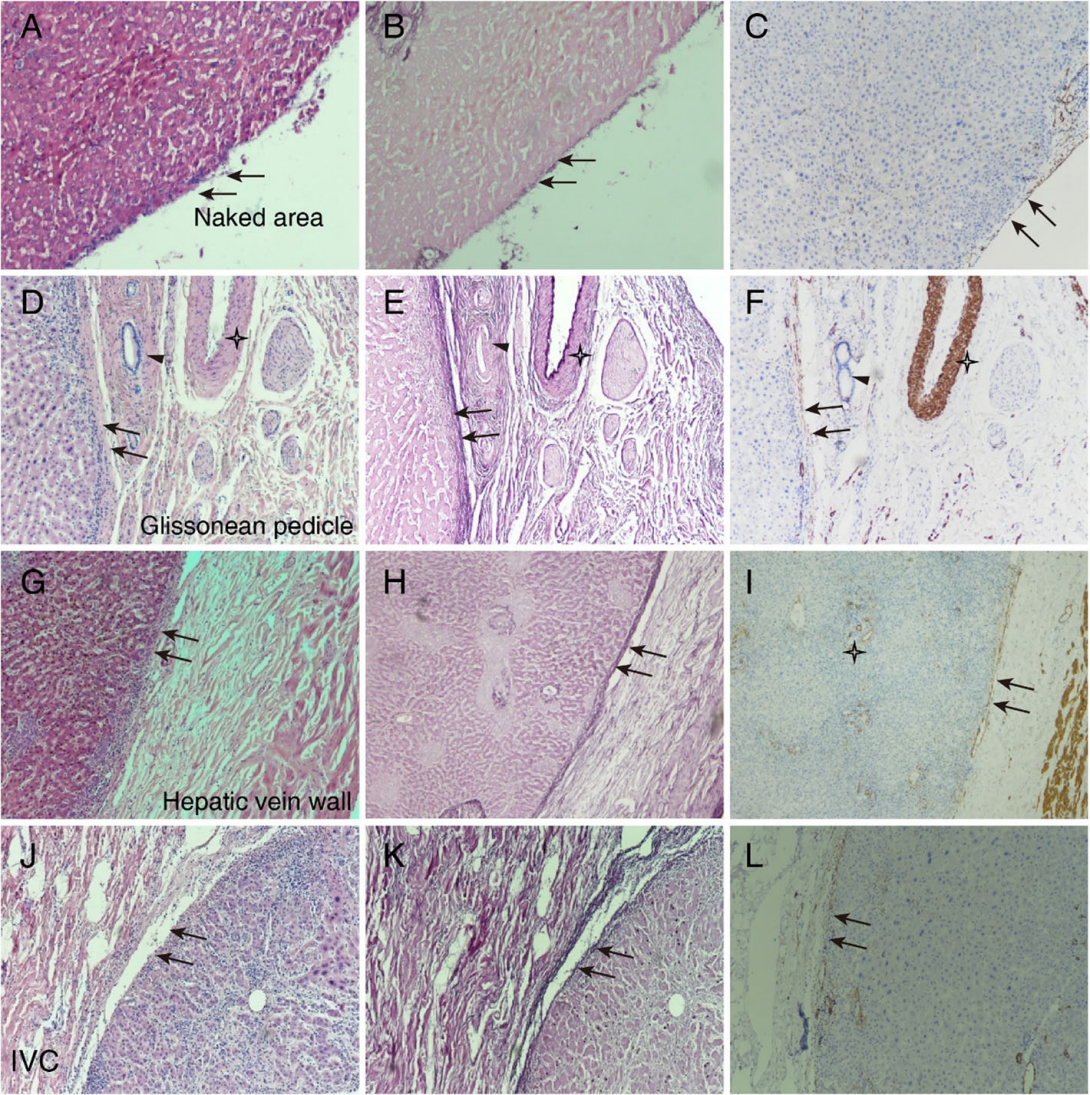
Histological findings of Laennec’s capsule in the liver Tissues in different areas were subjected to hematoxylin and eosin (**h**&**e**) (**a**, **d**, **g**, **j**), resorcinol-fuchsin (**r**&**f**) (**b**, **e**, **h**, **k**), and smooth muscle actin (SMA) staining (**c**, **f**, **i**, **l**), such as the naked area (**a**-**c**), and around the Glissonean pedicle (**d**-**f**), hepatic vein wall (**g**- **i**), and inferior vena cava (IVC) (**j**-**l**). The H&E staining results of the naked area, Glissonean pedicle, hepatic vein, and IVC are shown in (**a**), (**d**), (**g**), and (**j**). The thin fibrous layer on the surface of the liver parenchyma marked with arrows is Laennec’s capsule. The R&F staining results of the naked area, Glissonean pedicle, hepatic vein and IVC are shown in (**b**), (**e**), (**h**), and (**k**). The elastic fibers are stained light violet (arrows). SMA staining results of the naked area, Glissonean pedicle, hepatic vein, and IVC are shown in (**c**), (**f**), (**i**), and (**l**). Laennec’s capsule was positive for SMA staining at the different sites (arrows). Laennec’s capsule was observed on the naked area (**a**-**c**), and around the Glissonean pedicle (**d**-**f**), hepatic vein wall (**g**-**i**), and IVC (**j**-**l**). F and I show the artery and thin branches of the hepatic vein (asterisks). The immunoreactivity was positive not only in the arterial wall, but also in the vascular endothelium of the thin branches of the hepatic vein (asterisks). All staining images were taken at 100× magnification.

**Fig. 2:**
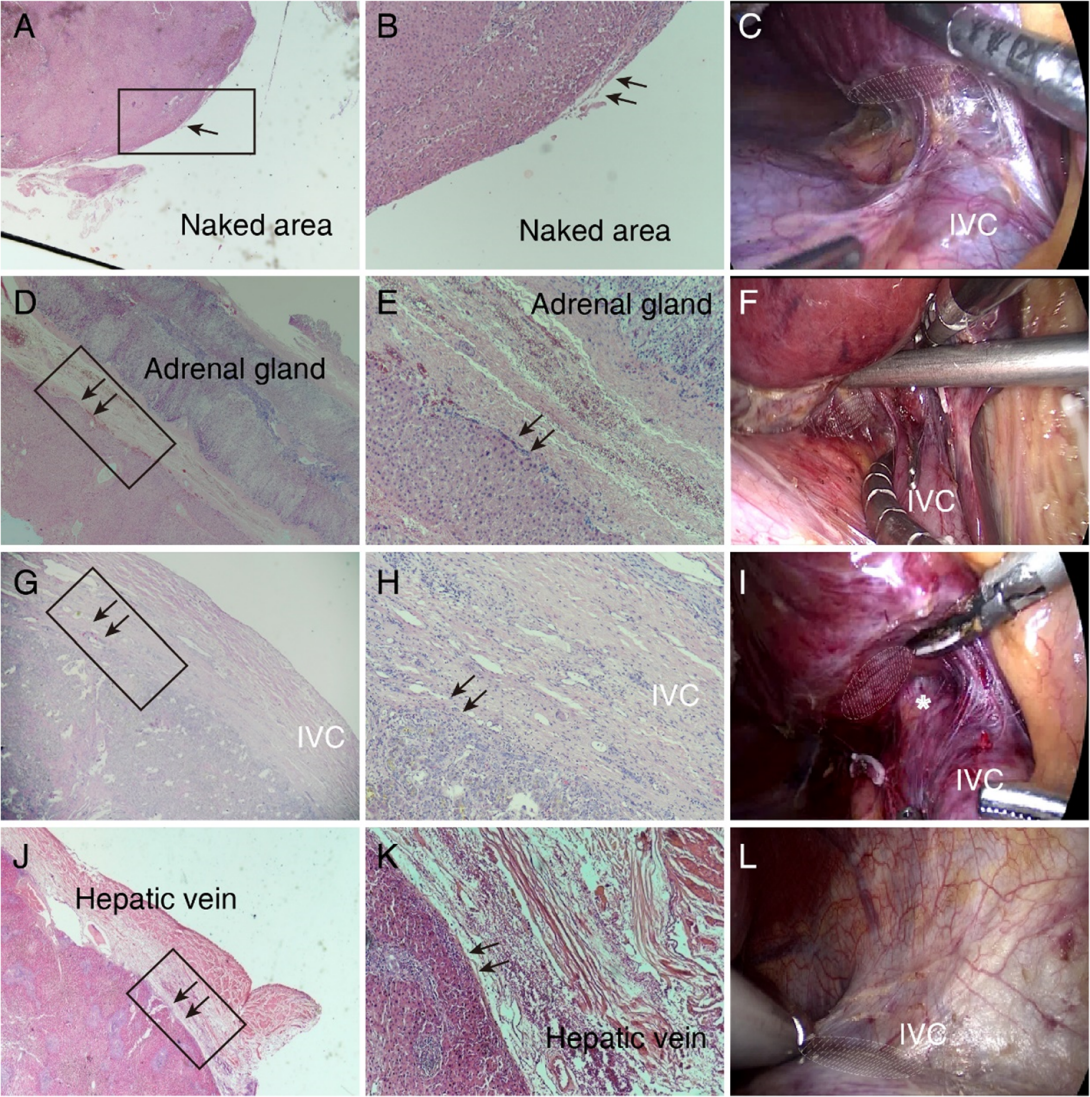
Liver mobilization with Laennec’s approach for laparoscopic anatomic hepatectomy (LAH) The surgical sites are shown under laparoscopy and microscopy when the liver was mobilized with Laennec’s approach (arrows). Laennec’s capsule and the adjacent tissues are shown by H&E staining in the naked area (**a**, **b**), adrenal gland (**d**, **e**), IVC (**g**, **h**), and hepatic veins (**j**, **k**). Representative laparoscopic views during liver mobilization are shown in the naked area (**c**), adrenal gland (**f**), IVC (**i**), and hepatic veins (**l**). And the Laennec’s capsule is marked with the elliptical shadow. (**a**), (**d**), (**e**), and (**j**) and (**b**), (**e**), (**h**), and (**k**) are the views under 20× and 100× magnification, respectively. The latter is a higher magnification view of the squares in the former.

We focused on the elastic fibers, which are known to be distributed in the serosal membrane of the viscera, to histologically confirm the presence of Laennec’s capsule. H&E and R&F staining are commonly used for elastic fibers. Laennec’s capsule was observed as a thin fibrous layer on the surface of the naked area (Fig. 1a, b). A similar fibrous layer was observed on the outside of the Glissonean pedicle (Fig. 1d, e), as a lining of the hepatic veins (Fig. 1g, h) and the IVC wall (Fig. 1j, k). Laennec’s capsule was also observed close to the adrenal gland (Fig. 2). To a greater or lesser degree, the major hepatic veins and IVC carried elastic fibers around their walls. These elastic fibers were usually thinner and less wavy than those of the liver capsule (Figs. 1, 4). Moreover, Laennec’s capsule was continuous with the liver capsule near the venous terminal.

We also considered the smooth muscle configuration to understand the configuration of the elastic fibers, because both usually coexist in the vascular wall and the subperitoneal connective tissues. The SMA immunohistochemical staining of Laennec’s capsule was positive at the different sites (Fig. 1c, f, i, l). In contrast, the two bile ducts around the Glissonean pedicle contained neither smooth muscle nor elastic fibers (Fig. 1d, e, f, arrowheads). We observed a positive result not only in the arterial wall but also in the vascular endothelium of the thin branches of the hepatic veins (Fig. 1f, i).

In summary, H&E and R&F staining showed that capsule packaging was present in the whole parenchyma of the naked area, Glissonean pedicle, hepatic veins, adrenal glands, and IVC. The fibrous structure of the liver capsule was denser compared with Laennec’s capsule. The liver capsule contained more elastic and collagen fibers. The capsule stained positively for SMA.

### The natural gap between Laennec’s capsule and the adjacent tissues at different sites

Based on the results shown in Fig. 1, a natural gap was observed between Laennec’s capsule and the adjacent tissues at different sites, such as Glissonean pedicles, the hepatic vein, and the IVC. The gap size varied from the sites, even within the same tissue. The gap between Laennec’s capsule and the corresponding adjacent tissues was measured in 10 samples. The gap size was 20–50 μm. The average gap size between Laennec’s capsule and the Glissonean pedicle was 32 ± 8.7 μm, while those between Laennec’s capsule and the hepatic vein and IVC were 26 ± 6.3 and 29 ± 7 μm, respectively. No significant difference was observed in the gaps among the sites.

### Liver mobilization using Laennec’s approach for a laparoscopic anatomic hepatectomy

The liver was mobilized and freed from adjacent ligaments and organs after the ligaments surrounding the liver were dissected. It was challenging to mobilize the liver in the naked area and the adrenal glands from the hepatic port under laparoscopy. Pathological staining showed that there was a natural gap between Laennec’s capsule and the adrenal glands, IVC, diaphragm, and the confluence of the hepatic veins (Fig. 1A and J). Therefore, we isolated the ligaments and tissues close to Laennec’s capsule after the visceral peritoneum was dissected above the adrenal gland, IVC, diaphragm, and the confluence of the hepatic veins (Fig. 2 and Additional file 1). The liver was mobilized by Laennec’s approach, which guided the surgeons to mobilize the liver in the correct gap and protect the adjacent tissues.

### Isolating the Glissonean pedicle using Laennec’s approach for laparoscopic anatomic hepatectomy

**Fig. 3:**
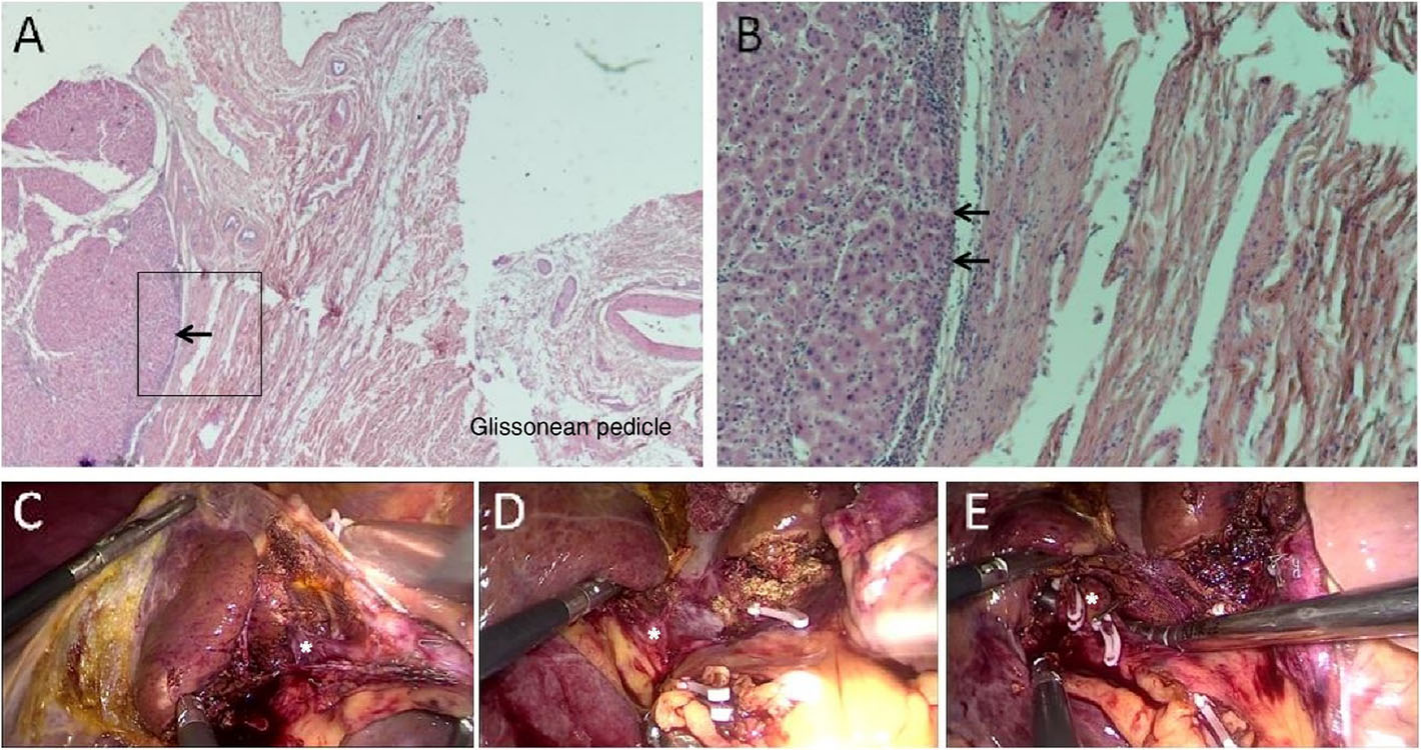
Isolating the Glissonean pedicle with Laennec’s approach for laparoscopic mesohepatectomy Laennec’s capsule was present at the parenchymal walls surrounding the hepatic portal, and the gap was clear. (**a**) and (**b**) are the 20× and 100× magnified views, respectively. (**b**) is a higher magnification view of the square in (**a**). Representative laparoscopic views during Glissonean pedicle isolation are shown for S4b (**c**), S1 pp. (**d**), S5, and S8 (**e**), which are marked with asterisks; Laennec’s capsule was located according to the elliptical shadow.

Although the Glissonian approach is the general strategy for hepatectomy, there are no clear anatomical guidelines for this approach [9]. There is a natural gap between Laennec’s capsule and the Glissonean pedicle, which we exposed in the hilus hepatis after dissecting the visceral peritoneum. The second and third Glissonean pedicles were isolated close to Laennec’s capsule (Fig. 3 and Additional file 2). A representative case underwent laparoscopic mesohepatectomy, where the Glissonean pedicles for Segment IV (S4), Segment Ipp (S1 pp), Segment V (S5), and Segment VIII (S8) were isolated using Laennec’s approach for a laparoscopic anatomic mesohepatectomy (Fig. 3 and Additional file 2). After the hepatic pedicles were dissected, Laennec’s capsule was exposed and marked with the elliptical shadow.

### Isolating the hepatic vein with Laennec’s approach for laparoscopic anatomic hepatectomy

Hepatic veins serve as intrahepatic landmarks for anatomic hepatectomy because they are the boundaries of segments or lobes [10]. However, it is challenging to safely isolate and expose the hepatic veins due to the increased risk of hemorrhage and hemostasis. There is also a natural gap between Laennec’s capsule and the hepatic veins. First, the main trunks of the hepatic veins were exposed and pursued to their roots. Golden finger can be gently slid along the upper surface of the veins with Laennec’s approach for LAH. The RHV or MHV serves as the intrahepatic landmark for an anatomic right posterior hepatectomy, or right or left hepatectomy.

**Fig. 4:**
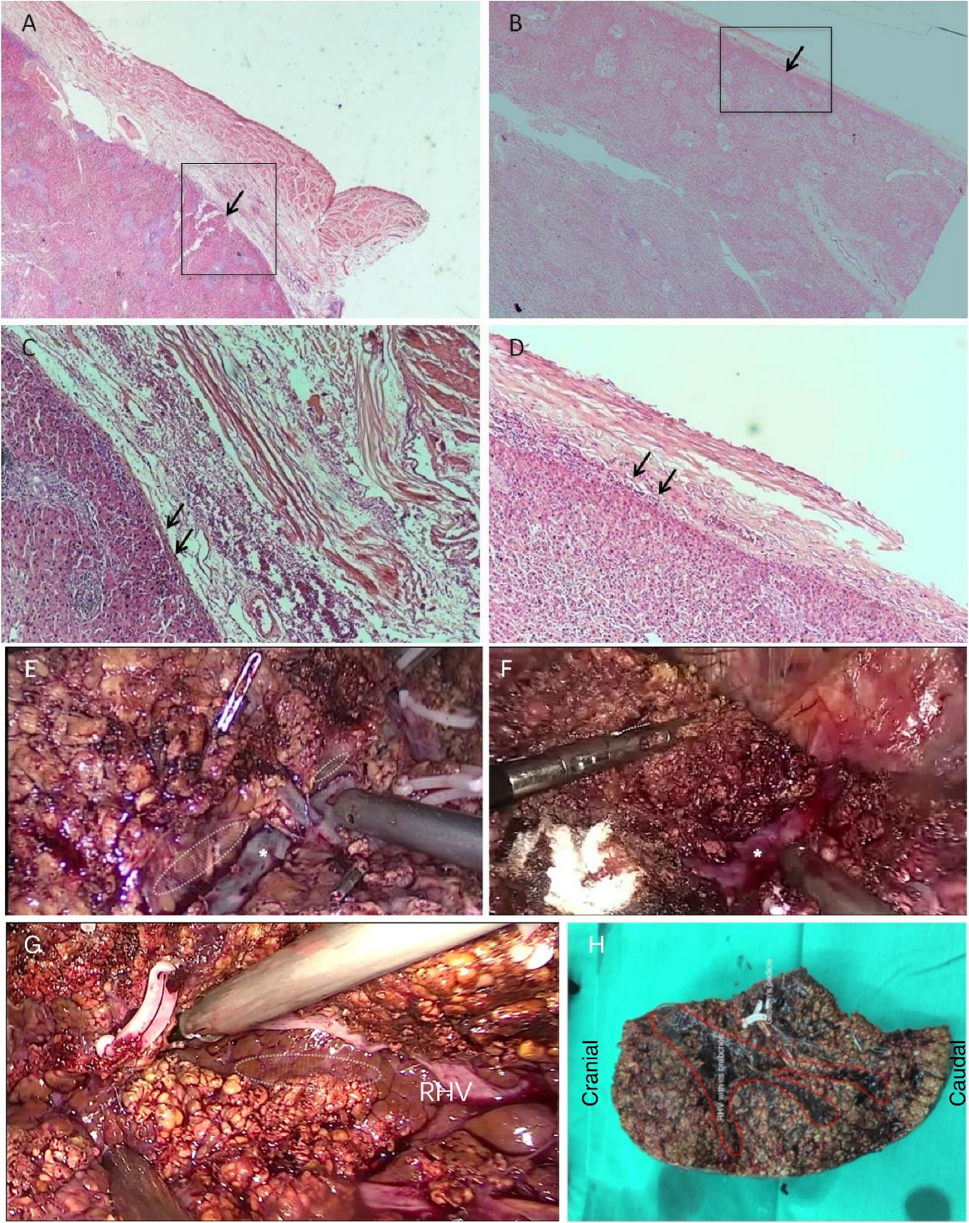
Isolating the hepatic vein with Laennec’s approach for LAH Laennec’s capsule and the middle hepatic vein are shown by H&E staining (**a**-**d**). (**a**) and (**b**) depict the venous onset and terminal at 20× magnification. (**c**) and (**d**) show a higher magnification view of the square in (**a**) and (**b**). Representative views during isolation of the hepatic vein are shown of the middle hepatic veins and their trunks during a right hepatectomy (**e** and **f**), in which Laennec’s capsule was located according to the elliptical shadow. Representative views during isolation of the right hepatic vein and its trunk are shown during a right posterior hepatectomy, in which Laennec’s capsule was located according to the elliptical shadow (**g**). The contour for the right hepatic vein is marked with a red line (**h**).

A representative case underwent a laparoscopic anatomic right hepatectomy, and the trunk of the MHV was isolated and exposed with Laennec’s approach for LAH. The parenchyma above the MHV was transected close to Laennec’s capsule (Additional file 3). The other case underwent laparoscopic anatomic right posterior hepatectomy to isolate and expose the branches and trunk of the RHV with Laennec’s approach for LAH (Fig. 4g). The parenchyma distal to the RHV was transected close to Laennec’s capsule. The hepatic veins for S6 and S7 were exposed and dissected.

### Hanging maneuver with Laennec’s approach for laparoscopic anatomic hepatectomy

**Fig. 5:**
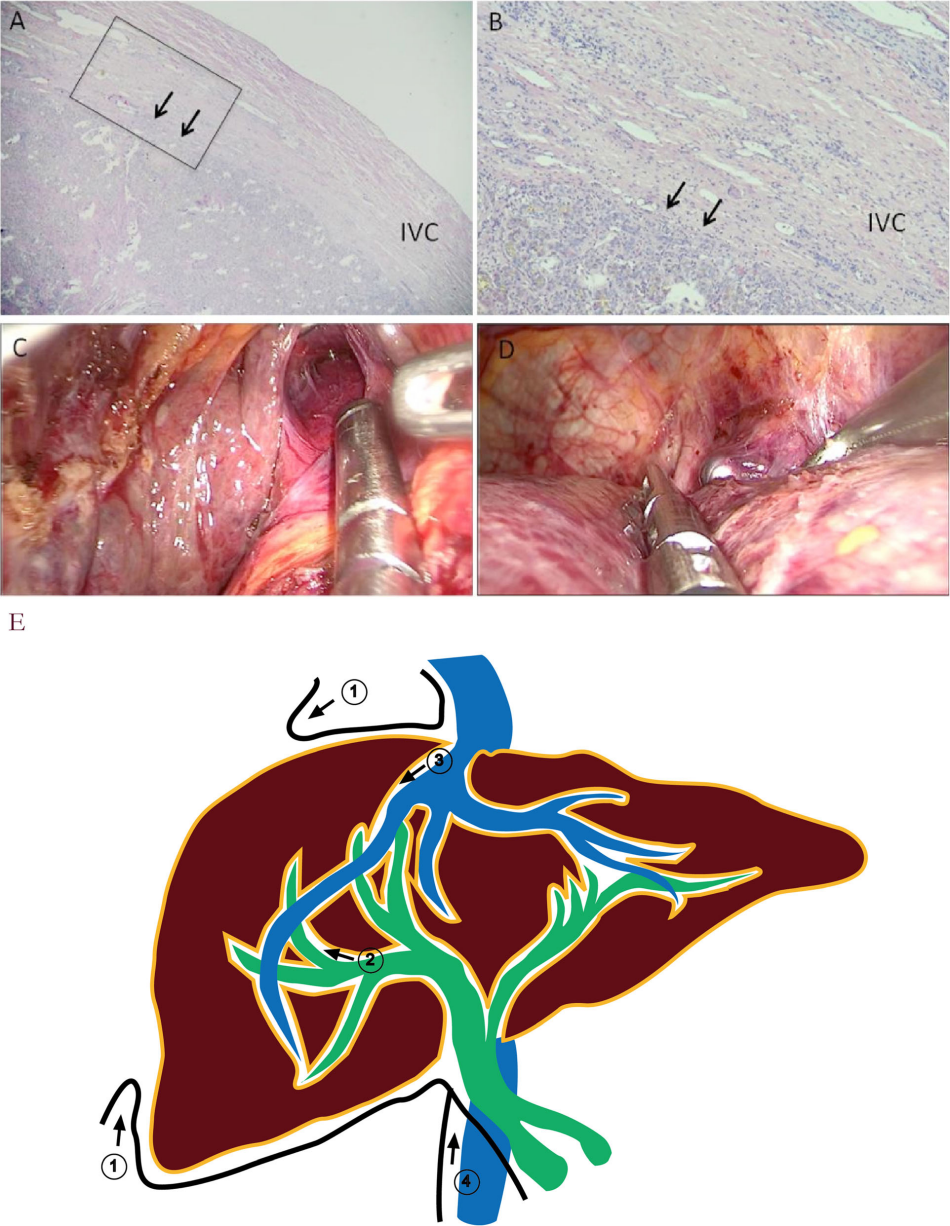
Hanging maneuver with Laennec’s approach for LAH Laennec’s capsule with H&E staining around the IVC was located with the arrow in (**a**) and (**b**). A thick elastic lamina is observed covering the entire IVC, with a gap (arrows). (**a**) and (**b**) are views under 20× and 100× magnification, respectively. (**b**) is a higher magnification view of the square in (**a**). (**c**) and (**d**) show the Hanging maneuver. (**e**) Sketch map of Laennec’s capsule and Laennec’s approach for hepatectomy. Sketch map of Laennec’s capsule is referred to Sugioka’s study in 2017. Laennec’s capsule is marked with a brown line and was observed as a continuous dense fibrous layer surrounding the whole liver independent of the serosa, Glissonean pedicle, cystic plate, and hepatic veins. The well-defined gaps between Laennec’s capsule and the surrounding tissues were identified after dissecting the visceral peritoneum. Laennec’s approach for a hepatectomy includes mobilizing the liver, isolating the Glissonean pedicle and hepatic vein, and performing Hanging maneuver. These steps were numbered sequentially.

Hanging maneuver is a practical strategy for the anterior approach to hepatectomy [11]. Many short hepatic veins and ligaments surround the IVC. Therefore, it is challenging to safely set up a tunnel along the IVC because of the risk of hemorrhage and gas embolization [12]. There is a natural gap between Laennec’s capsule and IVC. First, the visceral peritoneum was dissected, and the tunnel was set up to the root of the hepatic vein along the natural gap between Laennec’s capsule and the retrohepatic vena cava after dissecting several short hepatic veins(Fig. 5a-d and Additional file 4).

### Outcomes of the patients undergoing LAH with Laennec’s approach

In total, 84 cases underwent LAH with Laennec’s approach, including 26 for a right or posterior lobectomy, 26 for a segmentectomy, and 32 for a left hepatectomy. Laennec’s approach served to mobilize the liver, isolate the Glissonean pedicle and hepatic vein, and allow performance of the Hanging maneuver. The operation time was 277.23 min, and four cases were converted to an open hepatectomy for bleeding. The mean of hospital days was 9.80. Four cases had bile leakage, which resolved after 1 month. Therefore, it was feasible and efficient to use Laennec’s approach for LAH.

## Discussion

In summary, we confirmed the natural gap between Laennec’s capsule and the adjacent tissues with H&E, R&F, and SMA staining. This natural gap was considered as the anatomic landmark aiding exposure of the Glissonean pedicle and hepatic veins, mobilization of the liver, and performance of the Hanging maneuver during LAH. Our group firstly applied Laennec’s approach to LAH, and standardized the critical steps, such as isolation of the Glissonean pedicle and the hepatic veins, mobilization of the liver, and performance of the Hanging maneuver. Based on the outcomes of the 84 cases undergoing LAH, Laennec’s approach was feasible and efficient for LAH.

The extrahepatic and intrahepatic capsules are controversial. Walaeus first described the vasculo-biliary sheath, which contains the portal vein, the hepatic artery, and a bile duct, in 1640. The Glissonean pedicle was reported by Glisson in 1642. In 1802, Laennec first described how a capsule is a distinct structure from the serosa and Glissonean pedicles. Couinaud also confirmed Laennec’s capsule, which has no continuity with the Glissonean pedicle [13]. Hayashi et al. demonstrated that Laennec’s capsule surrounds the pedicles, hepatic veins, and IVC, and extends to the peripheral Glissonean pedicles and hepatic veins [14]. And Sugioka et al. firstly reported Laennec’ s capsule served as the landmark to isolate extrahepatic Glissonean pedicle [7]. We further confirmed Laennec’s capsule histologically, as shown in Fig. 1. Laennec’s capsule was observed as a continuous, dense fibrous layer surrounding the entire liver independent of the serosa, Glissonean pedicle, cystic plate, and hepatic veins. The well-defined gaps between Laennec’s capsule and its surrounding tissues were identified and entered into without parenchymal destruction after dissecting the visceral peritoneum. Knowledge of Laennec’s capsule is essential for understanding the comprehensive surgical anatomy of the liver. Herein, we firstly propose Laennec’s approach for LAH, as summarized in Fig. 5e. Sugioka et al. had just proposed the possibility of liver surgical anatomy based on Laennec’s capsule and just focused on the intrahepatic parenchyma surrounding the Glissonean pedicles. We put this idea into action and applied into comprehensive surgical anatomy of the liver based on Laennec’s capsule in four different sites including the naked area, Glissonean pedicles, hepatic vein and IVC.

Anatomical hepatectomy is the ideal operative strategy for liver neoplasms [15]. It is a critical procedure to mobilize the liver and isolate inflow and outflow. Laennec’s capsule serves as a special landmark for LAH, which can be referred to when isolating the Glissonean pedicle and hepatic veins from the liver parenchyma (i.e., the inflow and outflow). All extrahepatic Glissonean pedicles, including those of the caudate lobe, were isolated systematically according to our novel and comprehensive surgical anatomy of the liver based on Laennec’s capsule. This novel concept could also be applied to isolate and expose the main hepatic veins by preserving Laennec’s capsule up to the vein wall, mobilizing the liver, and performing the Hanging maneuver. The hepatic inflow and outflow were controlled during transection of the parenchyma. It is helpful for surgeons to follow up the continuous capsule around Glissonean pedicles or hepatic veins. Therefore, ALR was completely standardized via Laennec’s approach.

As a limitation, this study used an observational design and had no control group. We conclude that the approach used herein is feasible but not superior to other approaches. The outcomes were affected by the surgeon’s skill and experience and the patients’ anatomy and disease status. It will be challenging to investigate Laennec’s approach in a cohort study. In the near future, the approach will be extended to cover any type of liver resection. Future cohort studies should compare Laennec’s approach with other approaches for hepatectomy, and validate the outcomes.

## Conclusions

Our study illustrates that Laennec’s capsule, packaging of the whole liver independent of the adjacent tissues and intrahepatic vessels, served as the landmark for isolating Glissonean pedicle and hepatic veins, mobilizing the liver, and performing Belghiti maneuver. Laennec’s approach based on Laennec’s capsule contributes to standardization of the surgical technique for laparoscopic anatomic hepatectomy.

## Supplementary information

**Additional file 1.** Liver mobilization with laennec’s approach for laparoscopic anatomic hepatectomy.

**Additional file 2.** Glissonean pedicle isolation with laennec’s approach for laparoscopic anatomic mesohepatectomy.

**Additional file 3.** Hepatic vein isolation with laennec’s approach for laparoscopic anatomic hepatectomy.

**Additional file 4.** Belghiti maneuver with laennec’s approach for laparoscopic anatomic hepatectomy.

## Data Availability

All data generated or analyzed during this study are included in this published article and its supplementary information files. The datasets generated and analyzed during the current study are available from the corresponding author by email yudecai@nju.edu.cn on reasonable request.

## Abbreviations

ALR: Anatomical liver resection
H&E: Hematoxylin and eosin
HCC: Hepatocellular carcinoma
IVC: Inferior vena cava
LAH: Laparoscopic anatomic hepatectomy
R&F: resorcinol-fuchsin
SMA: Smooth muscle actin

## References

[1] Cho A, Yamamoto H, Kainuma O, Souda H, Ikeda A, Takiguchi N, Nagata M (2011). Safe and feasible extrahepatic Glissonean access in laparoscopic anatomical liver resection. Surg Endosc.

[2] Majno P, Mentha G, Toso C, Morel P, Peitgen HO, Fasel JH (2014). Anatomy of the liver: an outline with three levels of complexity--a further step towards tailored territorial liver resections. J Hepatol.

[3] Choi Y, Han H-S, Sultan AM, Yoon Y-S, Cho JY (2014). Glissonean pedicle approach in laparoscopic anatomical liver resection. Hepatogastroenterology.

[4] Wakabayashi G (2016). What has changed after the Morioka consensus conference 2014 on laparoscopic liver resection?. Hepatobiliary Surg Nutr.

[5] Couinaud C (1952). Segmental and lobar left hepatectomies. Journal De Chirurgie.

[6] Aoki S, Mizuma M, Hayashi H, Nakagawa K, Morikawa T, Motoi F, Naitoh T, Egawa S, Unno M (2016). Surgical anatomy of the right hepatic artery in Rouviere’s sulcus evaluated by preoperative multidetector-row CT images. BMC Surg.

[7] Sugioka A, Kato Y, Tanahashi Y (2017). Systematic extrahepatic Glissonean pedicle isolation for anatomical liver resection based on Laennec's capsule: proposal of a novel comprehensive surgical anatomy of the liver. Journal of Hepato-biliary-pancreatic Sciences.

[8] Yu DC, Wu XY, Sun XT, Ding YT (2018). Glissonian approach combined with major hepatic vein first for laparoscopic anatomic hepatectomy. Hepatobiliary Pancreat Dis Int.

[9] Machado MA, Surjan RC, Makdissi FF (2014). Intrahepatic glissonian approach for single-port laparoscopic liver resection. J Laparoendosc Adv Surg Tech A.

[10] Surjan Rodrigo C., Basseres Tiago, Pajecki Denis, Puzzo Daniel B., Makdissi Fabio F., Machado Marcel A.C., Battilana Alexandre Gustavo Bellorio (2016). A novel technique for hepatic vein reconstruction during hepatectomy. Journal of Surgical Case Reports.

[11] Mehrabi A, Mood ZA, Fonouni H, Kashfi A, Hillebrand N, Muller SA, Encke J, Buchler MW, Schmidt J (2009). A single-center experience of 500 liver transplants using the modified piggyback technique by Belghiti. Liver Transpl.

[12] Zhuo LW, Prasoon P, Wu H (2014). Role of vascular clamping in hepatic resection: a review. Hepatogastroenterology.

[13] Couinaud C (1954). Liver lobes and segments: notes on the anatomical architecture and surgery of the liver. Presse Med.

[14] Hayashi S, Murakami G, Ohtsuka A, Itoh M, Nakano T, Fukuzawa Y (2008). Connective tissue configuration in the human liver hilar region with special reference to the liver capsule and vascular sheath. J Hepato-Biliary-Pancreat Surg.

[15] Qiu D, Zhuang H, Han F (2017). Effect and influence factor analysis of intrahepatic Glisson’s sheath vascular disconnection approach for anatomical hepatectomy by three-dimensional laparoscope. J BUON.

